# Report of the Proceedings under a Brieve of Idiotry, (Peter Duncan against David Yoolow,) Tried at Coupar-Angus, 28-30 January, 1837; with an Appendix of Relative Documents, and Introduction

**Published:** 1838-07

**Authors:** 


### ART. IX.

*Report of the Proceedings under a Brieve of Idiotry,* (Peter Duncan *against* David Yoolow,) *tried at Coupar-Angus,* 28-30 *January,* 1837; *with an Appendix of relative Documents, and Introduction.* By LUDOVIC COLQUHOUN, Esq., Advocate.—*Edinburgh,* 1837. 8vo. pp. 116.

THE subject of this essay is of such importance to medical jurists, and is so ably treated by the author, that we deem it right to lose no time in laying an account of it before our readers. It is by monographs of this description that more real good is conferred on the science of medical jurisprudence than by the publication of the most voluminous treatises. The author is evidently master of his subject; and he treats the evidence given on both sides of this interesting case with such candour and liberality that it is impossible for either party to take offence from his remarks: but there is something more than this to recommend his introductory observations to the notice of our readers. They form not merely an introduction to the case reported, but they furnish a clear and concise history of the medico-legal relations of insanity, and of the relative value of general and medical evidence on these occasions. The witness, liable to be summoned on a commission of lunacy, will here discover the true bounds within which he should confine himself in delivering evidence, and the circumstances which will give to it the moral and legal force that every conscientious practitioner must desire it to possess. The author remarks, with great truth, in speaking of medical evidence—

"In many cases, its operation is most salutary; in some, though productive of loss of time and probably of patience, harmless; while in others its effect is positively mischievous; and instead of throwing light on the point to be ascertained, it envelops a clear case in doubt, and plunges a doubtful one into still deeper obscurity." (*Introduction,* p. xxxv.)

Most medical witnesses on these occasions are apt to consider that it rests with them to say whether a party against whom a brieve or commission is issued be insane or not. They thereby usurp the province of a jury; and, instead of expressing an *opinion* founded on facts, to be sifted and examined by the rules of common sense, they utter a *dictum,* which, in their estimation, ought to weigh down the evidence of ordinary witnesses, and even the observations made by the jury themselves on the mental condition of the supposed lunatic or idiot.

Let us, however, consider what kind of evidence is required of a medical witness examined on a commission. His evidence relates partly to matter of fact, and partly to matter of opinion: he states to the jury such facts relative to the question at issue as have fallen under his notice, or been elicited by the tests to which he has subjected the party. He states also the opinion which he deduces from these facts, and the reasons for that opinion. At the same time, the essential point for the consideration of the jury is not the opinion which the witness may have formed, but the facts from which it has been derived. But, although the verdict of a jury ought ultimately to be founded on their own inference from the facts proved in the case, the opinion of an intelligent and experienced medical witness is unquestionably well worthy their serious attention, as an aid to the formation of that inference; and therefore, in receiving the facts which he attests, the jury ought to consider the conclusion which he draws from them, and the reasons he assigns for having come to that conclusion. Should these reasons appear not to warrant his conclusion, while the jury reject the conclusion they still have the facts from which a more legitimate inference may be deduced by themselves. However eminent in his profession a witness may be, or however extensive his experience in relation to the subject of insanity, a jury ought in no case to adopt his opinion, unless the grounds of it are completely satisfactory to their own minds. Where these grounds are unsatisfactory, or where medical witnesses come to different conclusions on facts essentially the same, the jury must throw aside the opinions which they deem inconclusive, and form their own opinion from the facts actually established in evidence, as well as from their inspection and examination of the party.

With these prefatory remarks on the value of medical evidence, we shall extract from Mr. Colquhoun's Report a short account of the case of the individual who was the subject of this "brieve of idiotry." A brieve of fatuity or idiocy, according to the law of Scotland, relates to those persons only in whom there is a *total* incapacity of mind. The issue which the jury have to try is, whether the party be "*incompos mentis, fatuus et naturaliter idiota*," and not to be trusted regarding the alienation of his property. The issue must be considered and answered by them "*in terminis;*" and, therefore, unless satisfied that the evidence amounts to proof of actual idiocy, they are not entitled to cognosce, i. e. to find the party an idiot.

From the general evidence offered, it appears that David Yoolow was, at the time of the commission, about fifty-four years of age,—that, from the age of ten, he had been affected with paralysis, which had rendered him a cripple and confined him during the greater part of his life (forty years) to the house. From the death of his father, he had been chiefly attended to by a sister, who died about six months before the issuing of the brieve. During the lifetime of his father, who was a farmer, he had taken no part in the management of the farm, and his farming affairs were entirely superintended by his sister after his father's death. In consequence of the paralytic attack, he had been removed from school during his boyhood, and no further instruction was attempted to be given to him. His mind was, therefore, in a most uncultivated state. His father and sister appear to have regarded him as incapable of attending to matters of property; for, at their deaths, they vested the property in trustees for his benefit, and did not directly bequeath it to him. After the death of his sister, the next of kin, the pursuer in this case, Peter Duncan, suspecting that, from the weakness and imbecility of David Yoolow, he would be liable to be imposed on in the disposition of his property by designing persons, sued out this brieve, in order to have the actual state of his mind legally investigated. We need hardly remark further, that, if the party were cognosced, i.e. found to be an idiot, he would cease to have any person in law. He would be held to have been incapable of acting or of consenting to any act since the time at which his mental infirmity was declared to have commenced; and any act or deed done subsequently to that time, and before cognition, or which he might do after cognition, would be *ipso jure* null. The administration of his property and the care of his person would devolve thereafter upon a curator appointed by the Court; generally the next of kin. This curator or committee, as he is called in English law, is legally responsible for the conscientious exercise of the trust reposed in him.

The medical evidence as to the state of this person's mind was of a very conflicting nature. Several physicians appeared in support of the unsoundness of mind, among others Dr. Christison and Dr. Malcolm. Both of them argued that Yoolow was a man of unsound mind, unable to manage his affairs, and labouring under a great degree of imbecility. Five other medical witnesses deposed to the same effect, in language nearly similar. Not one, however, contended that there was absolute, but only partial, derangement of intellect; all were unanimous in considering him to be incapable of transacting business. The counsel for the claimant admitted, in his opening speech, that Yoolow was not absolutely bereft of reason; and, indeed, had not the admission been made, this would have been clearly established by the evidence adduced. The facts upon which these medical opinions were founded were closely investigated. Each witness was required to state the questions put by him to the party. For these and the answers we must refer to the work itself: we shall only observe, that many of the questions were answered with such shrewdness and sagacity as to leave the impression that the party answering them was scarcely a fit subject for the enquiry. Some of the questions were eminently absurd, and we may be well surprised how a sensible individual could have put them under the circumstances. Thus, Yoolow having stated that he had 1200*l.* in the bank, and that he received 20*l.* for interest, Mr. Symmons asked him how much that was the hundred (i. e. per cent.)? (p. 13); Yoolow said "he could not tell, and that he was no good hand at arithmetic." As his counsel, Mr. D. M'Neill afterwards remarked, (p. 80,) this "is a question not so easily answered even by some of those who by courtesy are termed learned." Indeed, the counsel actually put this question to one of the claimant's medical witnesses, in cross examination, (p. 24,) and he acknowledged that he was unable to answer it. What better or more sarcastic commentary could be offered on the absurdity of putting such a question to an alleged idiot!

Several non-medical witnesses were examined, and their evidence went to show that Yoolow was a person easily swayed or controlled by those about him; that he was of weak mind, guilty of many silly and unreasonable acts; that he had no shame or sense of propriety in regard to calls of nature before the servants, male or female, or before his sister, (p. 21;) that he required a servant to take care of him; and that he was accustomed to be humoured like a child.

On the other side, several medical witnesses stated that Yoolow was neither an idiot, fatuous, nor *non compos mentis:* they found nothing about him, from their observations, to indicate insanity or idiocy. All seemed to think that his attainments were limited, from want of experience and instruction only. Dr. Nimmo considered him to be a perfectly sane man,—a man of sound mind,—by no means an idiot or fatuous. (p. 35.) Mr. Lowe thought that he was of *sufficient* degree of soundness of mind to give directions to others to act for him; and that his being unable to act for himself was owing to decrepitude of body. (p. 42.) So far as we have examined the evidence, we cannot find that any of the witnesses on this side of the question, with the exception perhaps of Mr. Lowe, explicitly admitted that the defender was possessed of sufficient mental capacity to direct and manage his own affairs. They leave this to be inferred from their statements, that "he was not an *idiot*, but *quite the reverse*." (p. 30.) In a written opinion, signed by Dr. Nimmo and Mr. Bell, it is stated that Yoolow "possesses a strong though uncultivated mind; and is therefore perfectly competent to select proper persons to manage his affairs or to bequeath his property." (p. 33.)

A few respectable non-medical witnesses were examined; among others a clergyman, who found Yoolow well informed in the Scriptures, and able to return satisfactory answers when questioned on the principles of the Christian religion. So far from showing any trace of imbecility or weakness of intellect, this witness considered that the answers given to his questions "evidenced an average degree of information and intelligence." (p. 29.) Other witnesses deposed that, so far as their observation went, they thought him a person of sound mind: they saw nothing wrong about him. (p. 41.)

The jury having visited Mr. Yoolow, a number of questions were put to him in their presence, the answers to which were very correct and proper, (p. 109;) and the sheriff remarked, in summing up, that, from the ready and unembarrassed mode in which these answers were given, he felt satisfied at the time that the character of idiocy did not apply to him. After having stated the law on the subject, and represented to the jury that idiocy applies only to those who are entirely deprived of the faculty of reason, and in whom there is a *total* incapacity, he proceeds to observe that, if the incapacity proved be only *partial*, the brieve of idiotry would be inapplicable to this case, and another and different remedy, provided by the law, must be sought for. The medical witnesses, he observes, differ widely among themselves as to the general conclusions at which they had arrived on the state of Yoolow's powers of mind. "This is somewhat difficult to account for, and is very apt to lead us to believe that so great a difference of opinion cannot arise out of their evidence as *medical* men; on the contrary, that it is a case in which non-medical men, and all persons of common sense, may be equally capable of judging." (p. 112.) The jury have to consider, whether the whole of the medical evidence in favour of the brieve amounts to a proof of fatuity or idiocy against Yoolow, or only to a partial derangement of the intellect, to which the brieve of fatuity and idiocy does not apply. It is for them to judge whether some of the questions put by the medical witnesses were or were not applicable to the party's limited state of knowledge and acquirements, and to deficiency of education.

The jury unanimously returned a verdict against the brieve, finding the prisoner not idiotic.

In commenting on this case, we must first of all separate the idea of bodily infirmity from mental incapacity. All agreed as to the state of disease or helplessness of the defender; but because an individual is helpless or bed-ridden, this is no ground for finding him *incompos* and incapable of directing or managing his own affairs. The evidence of mental incapacity in Yoolow's case does not therefore rest upon the fact of his labouring under corporeal infirmity, although it is well known that, from a severe attack of paralysis, the mind, if already developed, is apt to suffer; or, if undeveloped, is apt to have its capacity for acquiring knowledge either enfeebled or destroyed. The questions which it appears to us this case involves are simply: What was the actual state of this individual's mind? and what did the law require to establish the brieve? We shall separate the medical conclusions from the premises on which they were founded; and we do not hesitate to declare our opinion, from the *whole* of the evidence adduced, that the defender was a person of weak mind, labouring, in the language of Dr. Christison, under a *great degree of imbecility;* and that he was unable to manage and direct his affairs with that ordinary discretion necessary to protect an individual from the designs of artful persons. We, of course, consider all the witnesses to be worthy of belief. On the affirmative side, it was shown that Yoolow had been confined to his house for a period of forty years; that he required persons to look after him; that his habits before servants, and even his sister, were indecent; that he was under the control of servants; that he had no settled pursuit or occupation, if we except the reading of the Scriptures; that, although possessed of a farm, he had not undertaken any bargain or sale, and had intrusted its management to one who appears to have treated him like a child. From the medical evidence, he seems to have been easily excited and rendered irritable by trivial causes; and, judging more from his general conduct than from the answers returned to the questions put at these examinations, we have no hesitation in saying that we think there are strong proofs of his being a weak-minded and imbecile man. That he was wholly unfit to manage or direct affairs of property may perhaps be better derived from the conduct of those who must have known him well from birth, than from the observations of others whose opinions were founded on occasional visits and on the answers returned to their questions. In his father's settlement of property, "he is described as a person of weak mind, and, on that account, provided for merely as a helpless child, without any right of management or interference. The leases of the father, accordingly, are left entirely past Yoolow, and made descendible to the daughter. This is the expressed opinion of the father in 1805 and 1810; and, in 1834, Agnes Yoolow, the sister, takes the same course, and leaves the leases to trustees, under the obligation of supporting her brother." (p. 50.)

We do not find any satisfactory answers to these points in the defence. The counsel, Mr. D. M'Neill, devotes the greater part of a most able speech to a critical analysis of the medical evidence for the claimant; but he says little on the evidence of the non-medical witnesses, passing over the points to which we have adverted as eccentric habits, and justifying and excusing them by a reference to eccentricities and peculiar pursuits adopted or taken up by certain individuals. It is quite possible that some parts of the defender's conduct may be ascribed to eccentricity; but it seems to us equally certain that other parts can only be set down to mental incapacity. It would, perhaps, require a nice judgment to draw the line between an act depending on eccentricity and one depending on mental incapacity, if the signification of the word is to be thus extended; but the true question really is, whether, what are thus called eccentric acts are to be tolerated in an individual, when the result may be injury to his own civil rights, or the rights of his family. Evidence, it is true, was adduced on the defender's side, which went to show that some persons "had known nothing wrong of him;" but this negative evidence amounts to very little when we consider that two of the witnesses spoke of the period of his boyhood, forty years before, and previous to the paralytic attack; while the other two, one of whom had certainly known him a long period, only met and conversed with him occasionally. It is not probable that such individuals were likely to observe those parts of his general conduct which could not escape the notice of others to whose care he was immediately intrusted. But the conduct of Yoolow's father and sister towards him appears to us a sufficient answer to evidence of this description. The defender's counsel refers this conduct to the *personal decrepitude* of Yoolow, and the *secluded life* he had led, (p. 72;) but, setting aside the expressed opinion of his father on the state of his son's *mind*, we cannot admit that these circumstances were sufficient to justify the total transference of property to trustees for his benefit, without allowing him the least right of management or interference. This, be it remembered, applies to a man at that time twenty-seven years of age; and, in 1834, the same course is followed by a sister towards a brother fifty-two years of age! We cannot consider that such a proceeding would have been adopted by these relatives towards one whose mind was, as described by some of the witnesses, active and *strong*, and who was capable of directing or managing his own affairs. We think this circumstance corroborates the views of those who appeared on the affirmative side of this question; and that it affords an answer to those who formed their notions of his having a directing and disposing mind from a few cursory examinations.

Having thus expressed our opinion as to the actual mental state of the individual, we have, lastly, to examine—What did the law require to establish the brieve, or what proof of mental incapacity or imbecility was demanded in order that this person should be deprived of the management of his property? Besides the issuing of a brieve of idiotry or insanity, there are other processes in the law of Scotland by which the property of a person, labouring under a *partial* incapacity may be protected. It is not our purpose to refer to these remedies: it may be sufficient to state that the interposition of the law is in them of a more lenient character, while the issuing of a brieve of idiotry or insanity is considered to be an extreme measure. Idiocy, it has been already remarked, implies, in the law of Scotland, a *total* defect of judgment, and an entire deprivation of the faculty of reason. As such, it could not be said that the subject of this brieve was an idiot. His mental incapacity was proved to be only *partial*, and he undoubtedly had, to a certain extent, the power of reason. Indeed, not one of the medical witnesses on the claimant's side asserted that this unfortunate being laboured under idiocy in its strict legal sense. Dr. Malcolm expressly stated that he was not an idiot. While, therefore, we allow, with Mr. Colquhoun, that Yoolow was not a fit subject for cognition under a brieve of idiotry, we perfectly agree with the witnesses for the claimant, that he was imbecile and a man of very weak intellect. Under these circumstances, we cannot be surprised at the verdict of the jury: their duty was of a very simple nature; they had merely to consider whether the mental incapacity of Yoolow was *partial* or *total*, and upon this point we think there cannot be a reasonable doubt.

We cannot take our leave of Mr. Colquhoun without again complimenting him on the manner in which he has executed a somewhat difficult task. We wish that his professional brethren would more frequently follow his example. There can be no want of materials; and, judging by the medico-legal knowledge displayed in this volume, we should say they would be thereby conferring a great benefit on the members of the two learned professions, and rendering an important service to society at large.

---

